# Non‐invasive assessment of superficial and deep layer circuits in human motor cortex

**DOI:** 10.1113/JP277849

**Published:** 2019-05-22

**Authors:** Alexander Kurz, Wei Xu, Patrick Wiegel, Christian Leukel, Stuart N. Baker

**Affiliations:** ^1^ Department of Sport Science University of Freiburg Freiburg 79117 Germany; ^2^ Bernstein Center Freiburg University of Freiburg Freiburg 79104 Germany; ^3^ Medical School Institute of Neuroscience Newcastle University Newcastle upon Tyne NE2 4HH UK

**Keywords:** laminar, TMS, monkey, human, motor control, sensory, nerve stimulation, sensorimotor decision

## Abstract

**Key points:**

- The first indirect (I) corticospinal volley from stimulation of the motor cortex consists of two parts: one that originates from infragranular layer 5 and a subsequent part with a delay of 0.6 ms to which supragranular layers contribute.
- Non‐invasive probing of these two parts was performed in humans using a refined electrophysiological method involving transcranial magnetic stimulation and peripheral nerve stimulation.
- Activity modulation of these two parts during a sensorimotor discrimination task was consistent with previous results in monkeys obtained with laminar recordings.

**Abstract:**

Circuits in superficial and deep layers play distinct roles in cortical computation, but current methods to study them in humans are limited. Here, we developed a novel approach for non‐invasive assessment of layer‐specific activity in the human motor cortex. We first conducted brain slice and *in vivo* experiments on monkey motor cortex to investigate the output timing from layer 5 (including corticospinal neurons) following extracellular stimulation. Neuron responses contained cyclical waves. The first wave was composed of two parts: the earliest part originated only from stimulation of layer 5; after 0.6 ms, stimuli to superficial layers 2/3 could also contribute. In healthy humans we then assessed different parts of the first corticospinal volley elicited by transcranial magnetic stimulation (TMS), by interacting TMS with stimulation of the median nerve generating an H‐reflex. By adjusting the delay between stimuli, we could assess the earliest volley evoked by TMS, and the part 0.6 ms later. Measurements were made while subjects performed a visuo‐motor discrimination task, which has been previously shown in monkey to modulate superficial motor cortical cells selectively depending on task difficulty. We showed a similar selective modulation of the later part of the TMS volley, as expected if this part of the volley is sensitive to superficial cortical excitability. We conclude that it is possible to segregate different cortical circuits which may refer to different motor cortex layers in humans, by exploiting small time differences in the corticospinal volleys evoked by non‐invasive stimulation.

## Key points

- The first indirect (I) corticospinal volley from stimulation of the motor cortex consists of two parts: one that originates from infragranular layer 5 and a subsequent part with a delay of 0.6 ms to which supragranular layers contribute.
- Non‐invasive probing of these two parts was performed in humans using a refined electrophysiological method involving transcranial magnetic stimulation and peripheral nerve stimulation.
- Activity modulation of these two parts during a sensorimotor discrimination task was consistent with previous results in monkeys obtained with laminar recordings.

## Introduction

Many modern approaches in the neurosciences aim to understand the brain at the level of functional microcircuits (Arber & Costa, 2018), which are sets of interconnected neurons defined by their anatomical, morphological and genetic characteristics. In the primary motor cortex (M1), circuits located in different layers appear to play different roles, reflecting their distinct anatomical connections. Circuits within supragranular layers appear crucial for processing task‐relevant sensory information (Thura & Cisek, 2014, 2016) and for learning (Peters *et al*. 2014, 2017b; Chen *et al*. 2015). By contrast, activity in infragranular layer 5 is correlated with the prevention and production of movement (Ebbesen & Brecht, 2017; Peters *et al*. 2017a; Soteropoulos, 2018). The study of M1 laminar circuits typically requires experiments in animal preparations. Previous attempts to resolve different cortical layers have used high‐resolution functional imaging in humans (Huber *et al*. 2017), but this requires measurements close to the maximal achievable spatial resolution and is governed by the sluggish BOLD response, making the method unsuitable for measuring rapid task‐related changes.

In this paper, we report the development of an electrophysiological approach offering the possibility of segregating different motor cortex circuits and layers in human M1. We begin with *in vitro* recordings from slices of M1 from macaque monkeys, a species with very similar circuits for neural control of movement to humans. These single cell recordings revealed a small temporal shift in the trans‐synaptic activation of layer 5 neurons following stimulation of deep *versus* superficial layers. We then confirmed *in vivo* that this activation time difference involves layer 5 corticospinal neurons; the resultant descending corticospinal volley was thus subtly delayed following stimulation of superficial compared to deep layers. The time scales (0.6 ms) of this difference are so small as to have no functional relevance, but the observation opens the intriguing possibility of inferring excitability changes of different circuits with non‐invasive methods in humans.

In humans, various methods exist to measure descending corticospinal volleys induced by transcranial magnetic stimulation (TMS) over M1. Some patient groups have epidural electrodes implanted over the cervical spinal cord. In these patients, it is possible to record corticospinal volleys directly (Di Lazzaro *et al*. 2008, 2018), but the invasive nature of the method makes it unsuitable for more general usage in healthy volunteers. A non‐invasive alternative is to interact a conditioning TMS pulse with a peripheral nerve test stimulus (PNS) which elicits a Hoffman (H)‐reflex (Nielsen *et al*. 1993; Taube *et al*. 2011, 2015; Leukel *et al*. 2012, 2015; Niemann *et al*. 2018; Wiegel *et al*. 2018). By changing the time interval between conditioning and test stimulus, a characteristic repetitive pattern of facilitation of the H‐reflex is observed reflecting the repetitive descending volleys generated by the conditioning stimulus. This approach has the advantage of assessing the descending volleys with a very high temporal resolution, as the time interval between stimuli can be set with arbitrary precision. In the present study, we investigated whether this non‐invasive approach may offer the possibility of probing different M1 microcircuits. Two different parts of the first corticospinal volley evoked by TMS were assessed: the earliest part of the first volley, and that 0.6 ms later. We hypothesized that the earliest part of the first volley originates from trans‐synaptic activation of fast conducting corticospinal output neurons in layer 5b by nearby cells within the same lamina (Nielsen *et al*. 1993, 1995; Nielsen & Petersen, 1995; Di Lazzaro *et al*. 2012, 2018). According to our results in the macaque monkey, 0.6 ms later there is the opportunity for superficial layer neurons to contribute to the activation of the corticospinal cells.

To test this hypothesis, we chose a task paradigm which has previously been demonstrated to modulate M1 in a layer‐selective manner. Chandrasekaran *et al*. (2017) recorded from neurons across different layers in monkey, while the animals were required to discriminate and respond to a visual stimulus displayed with varying levels of stimulus difficulty. Early during discrimination, neurons in superficial layers were more active for stimuli of low compared to high difficulty. By contrast, activity in lower layers (including layer 5) was unaffected by stimulus difficulty at this time. We therefore predicted that in humans performing this discrimination task, the earliest part of the first corticospinal volley evoked by TMS would be unaffected by stimulus difficulty, whereas the part 0.6 ms later would be greater when the subject was presented with an easy compared to a difficult stimulus. Our results are in good agreement with these predictions, and thus our approach potentially offers the opportunity for non‐invasive layer‐specific assessment of M1 function in human subjects.

## Methods

### Monkey *in vitro* experiments

All experimental procedures were carried out under the authority of personal and project licenses issued by the UK Home Office, were approved by the Animal Welfare and Ethical Review Board of Newcastle University (reference no. 423/15) and conform to regulations described in Grundy (2015). *In vitro* results were obtained from further analysis of layer 5 neurons obtained from a previous published study (Xu & Baker, 2018), in which full details of methods are given. Twelve female and six male rhesus macaques (*Macaca mulatta*) aged between 4 and 9 years were used. Animals were obtained from the Health Protection Agency UK and MRC Centre for Macaques UK. Food and water were given *ad libitum* until 12 h prior to surgery, when animals were fasted. All animal experiments were terminal.

Animals were initially sedated with intramuscular injection of ketamine (10 mg/kg) before general anaesthesia induction with either i.v. propofol (4 mg/kg) or inhaled sevoflurane (2.5% inhaled in O_2_). Animals were subsequently ventilated with 2.5–3.5% sevoflurane or desflurane in pure oxygen. Doses of i.v. buprenorphine (20 μg/kg) and meloxicam (0.3 mg/kg) were then given before head fixation into a stereotaxic frame. A continuous infusion of i.v. methylprednisolone (5.4 mg/kg/h) was given to prevent cerebral oedema and i.v. Hartman's solution (10 ml/kg/h) to maintain circulating volume. The animal's pulse oximetry, heart rate, and blood pressure were continuously monitored in order to gauge depth of anaesthesia. Core and peripheral temperatures were also measured, and body temperature maintained with both a heat blanket and a warm air circulation system. Tissue from the precentral gyrus of the primary motor cortex was removed via bilateral craniotomies after dousing exposed cortex with ice cold sucrose Ringer (concentrations in mM: 252 sucrose, 3 KCl, 1.25 NaH_2_PO_4_, 1 MgSO_4_, 1.2 CaCl_2_, 10 glucose, 24 NaHCO_3_, temperature 0–4°C). The medial border of the block of tissue was the sagittal fissure. The lateral border was approximately 2 cm lateral to the sagittal fissure. The anterior border was approximately 1 cm anterior to the central sulcus. The posterior border was just posterior to the central sulcus to include a small part of the primary somatosensory cortex for the purpose of orienting the brain slice. Brain tissue was sliced parasagitally at 450 μm thickness using a VF‐300 vibrating blade microtome (Precisionary Instruments LLC, Greenville, NC, USA) in ice cold sucrose Ringer solution. Slices were then transferred to and held in an interface chamber at room temperature containing artificial cerebrospinal fluid (ACSF, same constituents as sucrose Ringer solution apart from sucrose being replaced by 126 mM NaCl) bubbled with 95% O_2_ and 5% CO_2_. After M1 tissue removal animals were killed by transcardiac perfusion with ice‐cold Ringer solution and exsanguinated through the right atrium, as part of tissue collection for other unrelated experiments.

*In vitro* recordings were carried out in an interface recording chamber (model BSC‐ZT, Harvard Apparatus, Cambridge, UK) whilst superperfused with ACSF. Intracellular recording electrodes were pulled from borosilicate glass capillaries on a model P‐1000 Flaming–Brown puller (Sutter Instruments, Novato, CA, USA). Electrodes were filled with 2 M potassium acetate and 2% biocytin (Sigma‐Aldrich, Gillingham, UK) to achieve impedance between 100 and 150 MΩ. After recording, cells were filled with biocytin using repetitive current pulses (alternating 0.5 s‐long positive and negative square wave current injections at 0.2 nA for at least 20 min) and subsequently fixed in 4% paraformaldehyde and stained using a standard Vectastain ABC kit (Vector Laboratories, Peterborough, UK). Voltage recordings and current injections were carried out using a BA‐03X bridge amplifier (NPI Electronic, Tamm, Germany) with ×10 gain and low‐pass filter set to 10 kHz. Capacitance transients were compensated for and bridge balance was checked and corrected regularly. Custom‐made software (Collins & Baker, 2014) was used to move recording electrodes mounted on piezoelectric motors (NanoPZ Ultra‐High resolution actuator, Newport Corporation, Irvine, CA, USA). The software also monitored electrode voltage readings and injected currents through the recording electrodes (0.2–1 nA, 1 s in duration) via the digital and analog input–output functions of USB National Instruments data‐acquisition device (USB‐6356 X‐series, National Instruments, Austin, TX, USA). Data were captured using a Micro1401 interface (digitized at 25 kHz) and Spike2 software (Cambridge Electronic Design, Cambridge, UK).

Extracellular stimulation was delivered using a 16 parallel shank electrode (100 μm between contacts, A1x16 series from NeuroNexus, Ann Arboru, MI, USA) placed on the slice such that the row of shanks was perpendicular to the slice cortical surface and the first contact rested on the cortical surface. Stimuli delivered through the most superficial four contacts were deemed to be to layers 1 and 2 and those delivered through the 10th to 13th contacts were deemed to be to layer 5 according to prior histological studies (Matelli *et al*. 1985; Shepherd, 1998; Lacroix *et al*. 2004). Recording electrodes were targeted to layer 5 by aligning with the 12th contact of the stimulating electrode away from the cortical surface (and placed approximately 0.5 mm lateral to it). Biphasic stimuli (0.1 ms per phase, 20–100 μA, cathode leading, no time separation between phases) were delivered with an interstimulus interval of 100 ms through each contact in a pseudo‐random order using a custom‐made relay circuit and an isolated stimulator (model 2100, A‐M Systems, Ontario, Canada). Stimulus strength was typically increased from 20 μA (up to 100 μA) until either excitatory postsynaptic potentials (EPSPs) and/or action potentials were elicited by stimulating at least one electrode contact. Typically, 100–200 stimuli were delivered per stimulus site.

### Monkey *in vivo* experiments

One monkey was used in an *in vivo* experiment under anaesthesia to verify and extend the *in vitro* results. Anaesthesia induction was carried out with intramuscular ketamine (10 mg/kg) followed by i.v. propofol (4 mg/kg). Maintenance anaesthesia was established with sevoflurane (2.5% inhaled in O_2_) and alfentanil (0.2 μg/kg/min, i.v. infusion). Methylprednisolone (5.4 mg/kg/h i.v.) was given to prevent cerebral oedema. The animal was intubated and ventilated via a tracheostomy and a bilateral pneumothorax made to improve recording stability. Once all surgery was completed (see below), anaesthesia switched to i.v. infusions of ketamine (6 mg/kg/h) and midazolam (0.5 mg/kg/h), and the inhaled sevoflurane was gradually reduced to zero, as we have found that this regimen better preserves neuronal excitability. Neuromuscular block was achieved via atracurium (1.5 mg/kg loading dose followed by 0.75 mg/kg/h i.v.). Fluid balance was maintained by infusion of Hartman's solution (volume to bring total infusion rate to 10 ml/kg/h including drug solutions).

Continuous monitoring was carried out on the following parameters: heart rate, respiratory rate, end tidal CO_2_, inspired/expired sevoflurane, pulse oximetry, rectal and skin temperature, central arterial and venous blood pressure (via cannulae introduced through the neck vessels), and urinary output via a urinary catheter.

A craniotomy was made over the right M1 and a laminectomy was made to expose the left first thoracic spinal segment. A single shank 16‐contact electrode (150 μm between contacts, model A1x16 from NeuroNexus) was inserted into M1 in the precentral gyrus (18 mm lateral to midline in the hand representation) perpendicular to the cortical surface such that the most superficial contact was flush with the cortical surface. Stimulus current (0.2 ms, biphasic) was delivered through each contact in a pseudorandom order using a custom relay circuit and isolated stimulator, as described above. Five blocks of stimulus trials were carried out, each with at least 100 trials per stimulus contact. Each block used a different stimulus intensity (2, 2.5, 3, 4 or 5 mA in a randomized order) with an interstimulus interval of 100 ms. Spinal volleys were recorded using a pair of ball electrodes placed over the dorsum of the left spinal cord using an NL824 amplifier (gain 10k, bandpass 30 Hz to 10 kHz) and a Neurolog amplifier/filter system (Digitimer Ltd, Welwyn Garden City, UK). Data were captured at 25 kHz sampling rate using a Micro1401 interface and Spike2 software (Cambridge Electronic Design, Cambridge, UK).

At the end of the experiment the animal was given an overdose of i.v. propofol, and killed by exanguination and transcardiac perfusion with paraformaldehyde.

### Data analysis and statistics

For the *in vitro* recordings, neuronal input resistance and membrane time constants were measured and found to be in accordance with those previously published for primate and non‐primate pyramidal neurons (Connors *et al*. 1982; McCormick *et al*. 1985; Nowak *et al*. 2003; Chang & Luebke, 2007; Luebke & Chang, 2007). Input resistance was calculated from averaged small voltage deflections (<10 mV) to injections of hyperpolarizing current (1 s duration). Membrane time constants were calculated by measuring the gradient of the logarithm of the initial part of this voltage deflection.

Spikes were discriminated using Spike2 software and spike times were aligned by the peak of the intracellular action potential. In neurons where no action potentials were evoked, the size of the EPSP was measured by taking the difference between the peak mean voltage response evoked by a given stimulus contact and the mean voltage near the end of the trial (70 ms post stimulus). In neurons where action potentials were evoked, peri‐stimulus time histograms (PSTHs) were compiled from spike times in the first 10 ms post‐stimulus and the bin heights were normalized by dividing by the number of trials. These normalized PSTHs were averaged across all available cells.

To reveal high‐frequency oscillatory patterns in these population PSTHs more clearly, they were digitally bandpass filtered (400–2000 Hz), and the latency of the first peak in the filtered PSTH measured. We needed to test whether the latency of this first peak was significantly different between responses to superficial and deep stimuli. This was achieved by a Monte Carlo analysis as follows. The individual cell PSTHs from superficial and deep stimulation were randomly shuffled and arbitrarily assigned to two groups. These were averaged and high pass filtered as above, the first peak latency measured, and the difference between the latency in each group calculated. This was repeated 1000 times; the peak latency difference from the original data was compared with the distribution of the shuffled data, and used to estimate a *P* value for the Monte Carlo test.

All analysis was carried out using custom scripts written in the MATLAB environment (The MathWorks Inc., Natick, MA, USA).

### Human experiments

#### Subjects

27 healthy participants with no contraindications to TMS (Rossini *et al*. 2015) participated in two experiments (experiment 1: *n* = 13: 6 males and 7 females; 24 ± 2.4 years of age; experiment 2: *n* = 14: 9 males and 5 females; 25 ± 2 years of age). Five individuals (4 males, 1 female) participated in both experiments. All subjects were right‐handed according to the Edinburgh Questionnaire (Oldfield, 1971) and gave written informed consent to the procedures; the study was performed in accordance with the *Declaration of Helsinki* (latest revision in Fortaleza, Brazil) and approved by the local ethics committee in Freiburg (approval number 423/15).

#### Electromyography

Surface electromyogram (EMG) (EISA; Pfitec Biomedical Systems, Endingen, Germany) was recorded from the right flexor carpi radialis (FCR) and extensor carpi radialis muscles using bipolar surface electrodes (Blue Sensor P; Ambu, Bad Nauheim, Germany) placed 2 cm apart over the muscle belly. A ground electrode was placed at the caput ulnae. Impedance was below 5 kΩ. EMG signals were pre‐amplified (×100), further amplified (×2), bandpass filtered (10–1300 Hz) and sampled at 10 kHz.

#### Peripheral nerve stimulation

H‐reflexes were elicited with a constant current stimulator (DS7A; Digitimer) by stimulating the median nerve approximately 1–3 cm proximal to the elbow joint. Stimuli consisted of square wave pulses of 0.2 ms duration. The electrode arrangement was bipolar: a graphite‐coated rubber pad of 5 cm × 5 cm was used as anode and was fixed proximal to the olecranon. A custom‐made round pad (1 cm diameter) was used as the cathode and moved stepwise to detect the optimum position for eliciting H‐reflexes in the FCR. The optimum was defined as the site where low stimulation intensity (5–30 mA, monophasic pulse) elicited a consistent FCR H‐reflex with minimal M‐wave, and no H‐reflex in the antagonist extensor carpi radialis. After the optimum site was found, a self‐adhesive cathode (Blue Sensor P; Ambu) was fixed at this site.

#### TMS

Single‐pulse TMS was applied over the contralateral M1 wrist area using a Magstim 200^2^ stimulator with a BiStim unit (Magstim, Whitland, UK) and a 50 mm figure‐of‐eight coil. The handle of the coil was mounted on a stand that was positioned on top of the subject chair (Manfrotto Magic Arm; Lino Manfrotto, Cassola, Italy). TMS navigation (Brainsight 2; Rogue Research, Montreal, Canada) was used to monitor the position of the coil relative to the scalp to ensure that the set coil position remained the same throughout all stimuli.

The optimum site for evoking motor‐evoked potentials (MEPs) was determined by a mapping procedure. The coil was held tangentially on the scalp at an angle of ∼45° to the mid‐sagittal plane with the handle pointing laterally and posteriorly (inducing a PA directed current).

Resting motor threshold (RMT) was determined as the minimum stimulator output (as a percentage of the maximum stimulator output) required to evoke MEPs of ≥50 μV in at least three out of five consecutive trials applied at the same intensity (Rossini *et al*. 1994).

#### Conditioning of the H‐reflex by TMS

**Figure 1:**
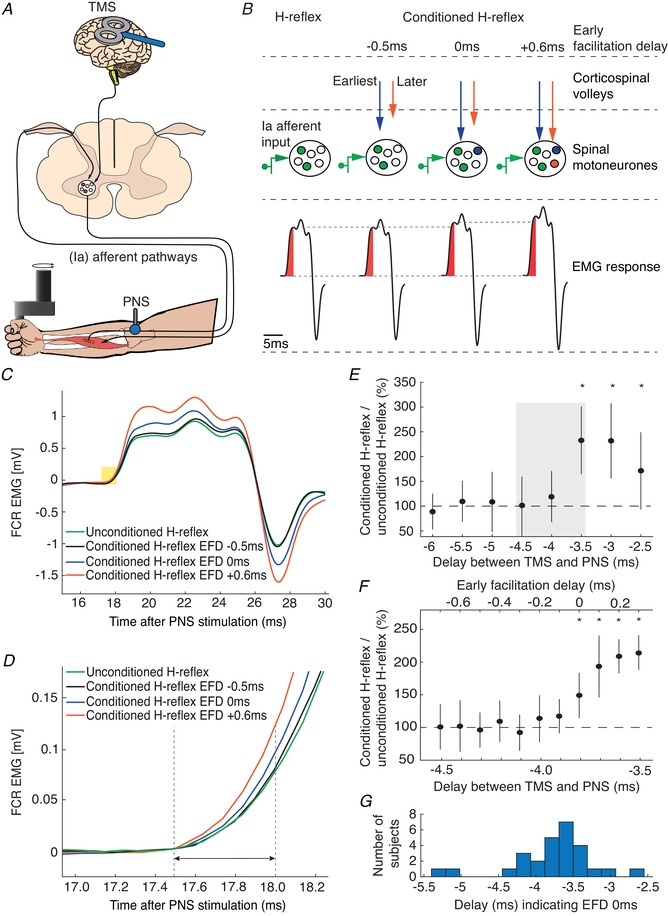
Conditioning of the FCR H‐reflex with TMS *A*, schematic representation of the experimental set‐up. *B*, principle of conditioning an H‐reflex evoked by PNS with TMS. TMS and PNS were applied together, so that TMS‐triggered volleys and the afferent volleys from PNS coincided at the spinal motoneurons. This leads to an increased recruitment of spinal motoneurons (middle part) and a corresponding increase in the size of the electromyographic response in the flexor carpi radialis (FCR) H‐reflex (lower part). We tested three different delays between TMS and PNS. At EFD −0.5 ms, the fastest conducting volley from TMS will not yet have arrived at spinal motoneurons at the time when the fastest conducting afferent volley from PNS arrives (blue and orange arrow). At EFD 0 ms, the fastest conducting corticospinal volley arrive at the same time at the spinal motoneurons as the fastest conducting afferent volley from PNS. At EFD +0.6 ms, subsequent volleys (orange arrow) arrive at the same time as the fastest afferent volley. *C*, mean traces of the unconditioned H‐reflex and of the conditioned H‐reflex at intervals EFD −0.5 ms, EFD 0 ms and EFD +0.6 ms. The yellow rectangle illustrates the 0.5 ms time window from H‐reflex onset used to quantify the H‐reflex facilitation. *D*, expanded view showing this time window in more detail. *E*, EFD 0 ms was determined by a two‐step procedure in each individual: we first tested delays between the application of TMS and the application of PNS from −5 ms to −2 ms, in steps of 0.5 ms (negative delays indicate that TMS was triggered after PNS). EFD 0 ms in this example was at a delay of −3.5 ms; conditioned H‐reflexes at this delay and at the subsequent delays were higher than unconditioned test H‐reflexes. The grey rectangle illustrates the time window tested in the second step of the procedure, shown in *F*. *F*, delays from −4.5 ms to −3.5 ms were again tested to denote EFD 0 ms in 0.1 ms steps (^*^ *P* < 0.05). *G*, the histogram illustrates the distribution of delays across all subjects between TMS and PNS indicating EFD 0 ms. [Color figure can be viewed at wileyonlinelibrary.com]

The objective of the conditioning technique is to promote coincidence at the spinal level of TMS‐evoked corticospinal volleys with afferent volleys elicited by PNS (Fig. 1 *A*). As shown schematically in Fig. 1 *B*, the median nerve stimulus alone recruits a fraction of the motoneuron pool (cells shaded in green), generating an H‐reflex. If TMS is delivered so that the fastest descending corticospinal volley (blue arrow in Fig. 1 *B*) reaches the motoneurons at the same time as the afferent input, more motoneurons may be discharged (‘0 ms’ in Fig. 1 *B*), leading to a larger, facilitated H‐reflex. Less negative delay between TMS and PNS allows more and more of the corticospinal volleys (orange arrow in Fig. 1 *B*) to influence the H‐reflex (see example in Fig. 1 *C* and *D*). The consecutive arrival of corticospinal volleys at the spinal level leads to temporal summation, resulting in a progressive increase of the H‐reflex.

For all measurements, electrical stimulation intensity was adjusted to evoke H‐reflexes of 15–25% of the maximum M‐wave (M_max_) (Crone *et al*. 1990), on the upward part of the H‐reflex/M‐wave recruitment curve. TMS was applied with an intensity of 120% of RMT, according to a recent study in which early indirect (I) volleys but no direct (D) waves could be evoked with intensities slightly above threshold using a 50‐mm figure‐of‐eight coil (Niemann *et al*. 2018). Stimuli were given at a repetition period of 5.5 s, to avoid post‐activation depression of the H‐reflex (Crone & Nielsen, 1989).

A two‐step procedure (rough followed by fine search) was performed to determine the time of coincident arrival of the fastest conducted peripheral and descending volleys (Fig. 1 *E*, *F*). In the first step (rough search, Fig. 1 *E*), delays between TMS and PNS were tested from −5 to −2 ms in steps of 0.5 ms, with 15 trials being recorded at each delay; in addition, 15 trials were recorded with PNS delivered alone. Note that negative delays indicate that TMS was triggered after PNS. Stimuli were delivered in 15 blocks. Each block consisted of eight trials testing all delays and an unconditioned H‐reflex in a randomized order. Paired Student's *t* tests were used to determine the first delay (starting with the most negative delay) where the conditioned reflex was significantly different from the unconditioned reflex (*P* < 0.05, uncorrected for multiple comparisons); this time was taken as our initial estimate of the earliest facilitation of the H‐reflex. In order to be accepted as the earliest facilitation, we required that the two subsequent less negative delays were also significantly higher than the unconditioned H‐reflexes. This criterion improved the robustness of the selection procedure, making it unlikely that the earliest facilitation was wrongly determined because of outliers caused by natural variability of the electrophysiological responses.

In the second step (fine search) of the selection procedure, we tested again different delays between TMS and PNS in steps of 0.1 ms in the time interval between the early facilitation of the first step and 1 ms later (11 steps in total) (Fig. 1 *F*). The same analysis was performed as in the first step of the procedure, allowing the earliest facilitation to be determined with 0.1 ms precision. The earliest facilitation resulting from the second analysis was designated the early facilitation delay (EFD) 0 ms. The distribution of the delays corresponding to EFD 0 ms across our subject population is illustrated in Fig. 1 *G*.

#### Dot motion discrimination task

**Figure 2:**
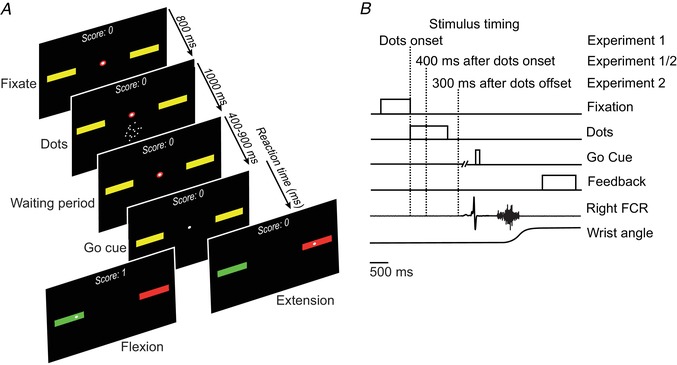
Dot motion discrimination task *A*, the display presented to the subject during task performance. *B*, the sequence of events in the dot motion discrimination task, showing the three points at which stimulation was delivered (dotted lines). [Color figure can be viewed at wileyonlinelibrary.com]

The task was a variant of the classical random dot motion discrimination task (Britten *et al*. 1992). Subjects reported the net direction of motion (left or right) in a random dot kinematogram by making wrist flexion and extension movements. The visual stimuli were generated using MATLAB and presented with 75 Hz frame rate on a 21‐inch LCD monitor with 800 × 600 pixels resolution positioned 40 cm away from the subject. We used the same algorithm and parameters as in a previous study in monkeys (Peixoto *et al*. 2018). The subjects used a robotic manipulandum, which tracked the wrist position and plotted it as a cursor on a computer screen, and made their choices by placing the cursor in a left or right target that was displayed on the screen. A trial began with the wrist in the neutral position, corresponding to the cursor in the screen centre between the two yellow targets; a visual fixation spot was superimposed on the cursor. After a fixation time of 800 ms, the random dot kinematogram was displayed for 1 s. After a waiting period (between 400 and 900 ms), the fixation dot disappeared and the subjects moved to indicate their decision. Upon completion of the movement, the correct target turned green, and the incorrect one red (Fig. 2 *A*). The subjects were rewarded if they hit the correct target with a 1 eurocent payment, paid as a total sum earnt at the end of the experiment. The robotic manipulandum then pushed the wrist back to the neutral position, and a new trial started 1.5–2 s later.

The random dot kinematograms were presented with two levels of difficulty, referring to the motion coherence of the visual cue. The difficulties were continually adjusted to yield 55% correct responses with the difficult (low coherence) cues, and 80% with easy (high coherence) cues.

#### Electrophysiological probing during the dot motion discrimination task

In two experiments, probing was performed at the onset of the visual cue and 400 ms afterwards (experiment 1), and 400 ms after visual cue onset and 300 ms after the onset of the waiting period (experiment 2, Fig. 2 *B*). Testing was split across two separate experiments in this way to avoid subject fatigue, as if conducted in a single session, completing the dot motion discrimination task alone would have taken more than 2 h. We recorded conditioned H‐reflexes at time intervals EFD 0 ms and EFD +0.6 ms and also recorded unconditioned test H‐reflexes in both experiments. Stimuli were applied in every trial. The delay between subsequent stimuli was always 5.5 s to avoid changes in post‐activation depression of the H‐reflex (Crone & Nielsen, 1989). All conditions were inter‐mixed and pseudo‐randomized. Twenty trials were recorded for each condition.

#### Data analysis and statistics

Root‐mean‐squared (RMS) values of the initial 0.5 ms from H‐reflex onset were calculated from the unrectified FCR EMG as described in Wiegel *et al*. (2018). The H‐reflex onset was visually determined in each subject based on the plot of superimposed test H‐reflexes from all trials. Before calculating RMS values, in each trial we corrected for offsets of the baseline EMG by setting the value at H‐reflex onset to zero. The reaction time was defined as the time interval between the fixation point offset and an increase (>4 standard deviations above the baseline mean) in the rectified EMG activity of the responding muscle. Mean background EMG activity of 50 ms prior stimulation was calculated in every trial. Trials in which background EMG activity exceeded the mean pre‐stimulation EMG activity of all trials recorded during the initial delay identification procedure (resting condition) by more than 2 standard deviations were excluded from further analyses, ensuring that the results cannot be biased by even slight modulations of the background EMG. This procedure resulted in the exclusion of 6.6 ± 10.3 trials per subject (mean ± SD across all subjects). One subject (experiment 2) was excluded due to increased pre‐stimulation EMG activity in all trials.

H‐reflex facilitation was calculated as the percentage change of conditioned H‐reflexes compared to unconditioned test H‐reflexes (conditioned H‐reflex/unconditioned test H‐reflex × 100%). Cue‐related H‐reflex modulation for each stimulation timing (dot onset, 400 ms after dot onset, 300 ms after delay onset) and delay condition (EFD 0 ms and EFD 0.6 ms) was calculated as the percentage change of H‐reflex facilitation for easy cue trials compared to difficult cue trials (H‐reflex facilitation easy cue/H‐reflex facilitation difficult cue × 100%).

All datasets showed normality and homogeneity, tested by the Kolmogorov–Smirnov test and the Levene's test, respectively. Unpaired Student's *t* tests were performed when comparing results of experiment 1 and experiment 2; paired Student's *t* tests were performed for all other *a priori* and *post hoc* analyses. For conditioned H‐reflexes recorded during the dot motion discrimination task, pre‐planned comparisons were performed for flexion and extension cues, for experiment 1 and experiment 2 separately. The level of significance was set to *P* < 0.05 for all tests and adjusted for multiple comparisons with the Bonferroni correction. Note that raw *P*‐values are presented, and statements about the significance are made in the text.

Mean values and standard error of the mean (SEM) are reported. Data were statistically analysed with SPSS Statistics 24.0 software (IBM Corp., Armonk, NY, USA).

## Results

### Stimulation of superficial and deep cortical layers in monkey elicits small time differences in activation of deep layer neurons

**Figure 3:**
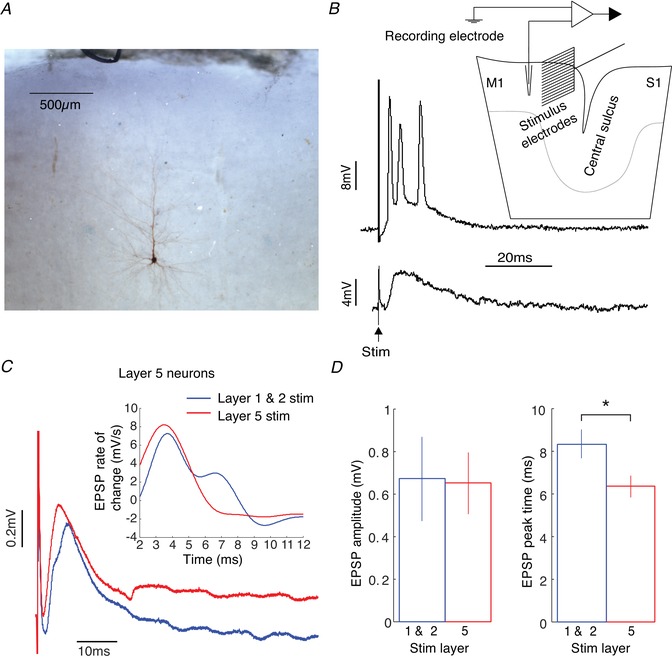
Intracellular responses of layer 5 neurons to extracellular stimulation *A*, labelled layer 5 pyramidal neuron. *B*, example of stimulus‐evoked spikes (upper part) and EPSP (lower part). Inset shows recording and stimulus set‐up. *C*, plot of mean EPSP waveform averaged across all layer 5 neurons in response to stimuli in layers 1/2 (blue) and layer 5 (red). Inset shows the first derivative of the EPSP waveform with respect to time for 2–12 ms post‐stimulus. *D*, comparison of mean evoked EPSP size and rise time evoked by layers 1/2 (blue) and layer 5 (red) stimulation (paired Student's *t* test, ^*^ *P* < 0.05). [Color figure can be viewed at wileyonlinelibrary.com]

Of 56 cells recorded from layer 5, four were filled with biocytin and successfully reconstructed histologically. All of these neurons were pyramidal cells (Fig. 3 *A*); as their electrophysiological properties matched the other (unreconstructed) cells in our dataset, we concluded that the vast majority of our recordings were likely to be from pyramidal neurons.

Stimulation with the 16‐contact probe electrode sometimes generated a subthreshold compound EPSP in the recorded layer 5 cell, and on other occasions the EPSP exceeded spike threshold and generated one or more action potentials; examples of these two possibilities are illustrated in Fig. 3 *B*. We analysed cells with or without spike responses separately.

Figure 3 *C* illustrates a grand average of all intracellular recordings with a subthreshold EPSP following cortical stimulation either nearby in layer 5 or in the more superficial layer 2/3 (32 cells). This average makes it clear that there was a small temporal shift (2.4 ms) in the EPSP peak between the two stimulation sites. The first derivative of the averaged EPSP waveform (Fig. 3 *C* inset) clearly shows that layer 5 stimuli produce EPSPs with a higher rise rate. Additionally, layer 1 and 2 stimuli produce EPSPs with an inflexion point at ∼6 ms, suggesting a later second excitatory input from another set of synapses. Figure 3 *D* presents measures of these responses made from individual cells. While there was no difference in the EPSP amplitude elicited by superficial and deep stimuli, the EPSP peak time was significantly later for superficial stimulation (difference between mean peaks is 2.0 ms, *P* < 0.05, paired *t* test, Fig. 3 *D*). This implies that EPSPs generated from superficial stimuli (that likely activate apical dendrites) will reach spiking threshold at the soma later than those from the deep stimuli (which likely activate basal dendrites). This corresponds to differing EPSP rise times elicited from synapses at different distances to the soma (Rall, 1967; Sjostrom & Hausser, 2006). The faster rise time of EPSPs evoked from layer 5 is better illustrated with the first time derivative of the averaged EPSP waveform (inset Fig. 3 *C*). The EPSP onset is superimposed onto the capacitive decay of the stimulus artefact which precludes precise measurements of onset times. However, the EPSPs evoked from layer 5 probably have a slightly earlier onset latency, and therefore the observed initial inflexion appears higher and earlier. There is also a later inflexion point at ∼5 ms in the layer 1 and 2 evoked EPSP suggesting a later excitatory input from another set of synapses.

**Figure 4:**
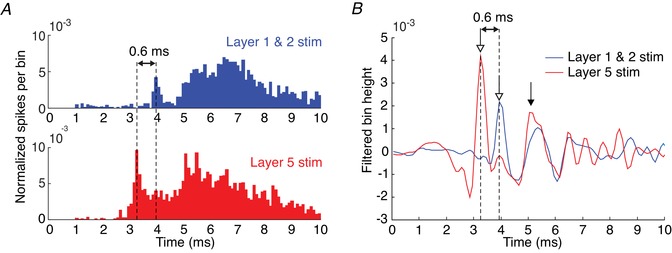
Population spiking response evoked by extracellular stimulation *A*, normalized peri‐stimulus histogram of layer 5 neurons’ spiking responses to stimulation in layers 1/2 (blue bars) and layer 5 (red bars). Bin heights represents spikes per bin per stimulus averaged across all layer 5 neurons tested with extracellular stimulation (bin width = 0.1 ms). *B*, bandpass filtered bin heights from panel *A* (400–2000 Hz). Arrows indicate peaks in the waveform: open arrows first peak; filled arrow second peak. [Color figure can be viewed at wileyonlinelibrary.com]

Our recordings also included cells which responded to the cortical microstimulation with spikes (24 cells); this allowed us to check whether the temporal shift in synaptic potentials shown in Fig. 3 *D* translated into an altered timing of evoked spikes (i.e. if superficial stimuli evoke spikes that occur later than deep stimuli). Figure 4 *A* presents averaged normalized peri‐stimulus time histograms, compiled separately for superficial and deep stimuli, for all spiking cells. The PSTH bin heights in Fig. 4 *A* were calculated by dividing by the number of stimuli for each neuron, then averaging across all neurons. Pooling across all cells mimics the population response from M1 to a single stimulus that activates either the superficial or the deep cortical layers. It is apparent that the first response peak also occurs slightly later for superficial stimulation. The quasi‐population response from layer 5 in Fig. 4 *A* will generate downstream spinal volleys *in vivo*. In order to simulate what the spinal field potential might look like from this population response, the PSTHs were digitally bandpass filtered in Fig. 4 *B* to simulate the filtering carried out by tissue (Telenczuk *et al*. 2011). These traces show a striking similarity to the timing of repetitive descending corticospinal volleys (I volleys) reported in both humans and animals following electrical and magnetic stimulation of the cortex (Patton & Amassian, 1954; Burke *et al*. 1993; Edgley *et al*. 1997; Ziemann & Rothwell, 2000; Di Lazzaro & Rothwell, 2014; Cirillo & Perez, 2015). However, importantly for the current study, the first peak (open arrows in Fig. 4 *B*) was 0.6 ms later following superficial compared to deep stimulation (*P* < 0.001, Monte Carlo test, see Methods). By contrast, the second peak (filled arrow, Fig. 4 *B*) was largely overlapping in time between the two stimulation locations.

### Time‐shifted activation from deep and superficial layer stimulation can also be observed in descending corticospinal volleys *in vivo*

**Figure 5:**
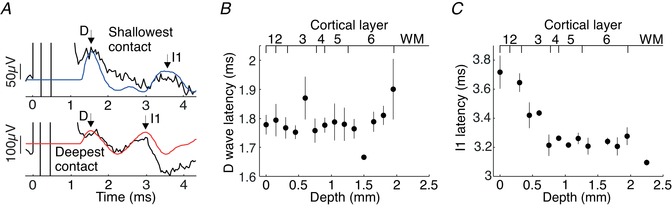
I1 shifts latency with changing depth of M1 cortical stimulation *A*, plot of averaged spinal volleys in response to stimulation through the shallowest (top) and deepest (bottom) electrode contact. Grey traces represent bandpass filtered volleys (400–2000 Hz). *B*, plot of average D volley latency against stimulus depth. Data averaged across 5 blocks of trials using 5 different stimulus intensities; error bars represent SEM.  *C*, as for *B*, but for the I1 volley. [Color figure can be viewed at wileyonlinelibrary.com]

Our *in vitro* recordings provide fine‐grain access to single cell discharge and its underlying synaptic mechanisms. As all cells were in layer 5, it is reasonable to expect that at least some of them projected to the corticospinal tract, but it was not possible to identify projection targets with certainty. We therefore checked in an anesthetized intact monkey whether the small time shift in activation which we observed *in vitro* translated to a time shift in the descending corticospinal volley. Figure 5 *A* presents a recording from the dorsal surface of the first segment of the thoracic spinal cord following cortical stimulation through a silicon probe with 16 equally spaced contacts. A direct (D) volley was elicited at short latency after the stimulus, with similar timing from the deepest and shallowest stimulation site. This presumably reflects some direct stimulus spread to the large, highly excitable corticospinal cells, including from the most superficial stimuli. It is well known that even electrical stimulation to the cortical surface can generate a D volley (Patton & Amassian, 1953). By contrast, the first indirect volley (I1) was clearly later from the most superficial stimulation. Latency measurements at all depths are presented in Fig. 5 *B* and *C*. While the D volley latency remained relatively constant independent of depth (*P* = 0.77, one‐way ANOVA), the I1 volley appeared around 0.5 ms later for the most superficial stimulation compared to deep stimuli (*P* < 0.01, one‐way ANOVA), which closely matches the time disparity of 0.6 ms between superficial and deep stimulus‐evoked spikes in the *in vitro* experiment. The cortical layers were estimated according to depth of electrode contact using previously published measurements of cortical layer depths for the macaque M1 (Shepherd, 1998). There was no significant difference in latencies across the five different stimulus intensities tested (2–5 mA) for all stimulus depths (*P* = 0.17, one‐way ANOVA). The interval between D and I1 waves is around 1.4–2 ms, which closely matches the value of ∼1.5 ms reported previously (Kernell & Chien‐Ping, 1967).

### In humans, only the later part of the first corticospinal volley is modulated by cue difficulty during visual perception

**Figure 6:**
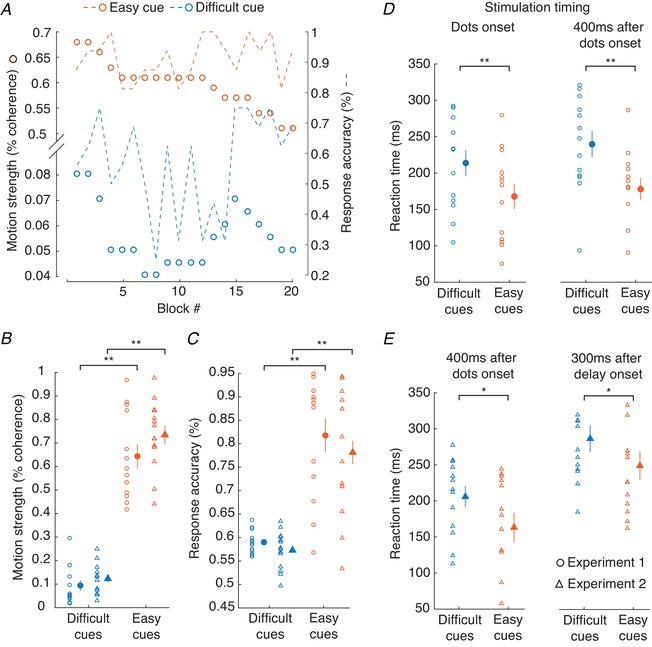
Behavioural results from the dot motion discrimination task *A*, adjustment of the motion coherence for difficult cues (blue dots) and easy cues (orange dots) in one subject over the time course of an experiment. Dashed lines indicate the corresponding response accuracy for difficult and easy cues, respectively. *B*, motion strength for difficult and easy cues (average of 20 blocks). The filled circles represent grand mean values ± SEM. *C*, the same for response accuracy. *D–E*, reaction times of the subjects following difficult and easy cues. *D*, stimulation given at dot onset (left panel) and stimulation given 400 ms after dot onset (right panel), in experiment 1. *E*, left panel, as right panel in *D*, but for experiment 2. Right panel, stimulation given 300 ms after delay onset. Filled circles and triangles represent grand mean values ± SEM of all subjects (^*^ *P* < 0.05, ^**^ *P* < 0.01). [Color figure can be viewed at wileyonlinelibrary.com]

In the dot motion discrimination task, the random dot kinematograms were presented with two levels of difficulty, referring to the motion coherence of the visual cue. The difficulties were continually adjusted to yield 55% correct responses with the difficult (low coherence) cues, and 80% with easy (high coherence). As subjects slowly became better at making the discrimination, motion coherence was continuously reduced throughout a testing session (see example in Fig. 6 *A*). Coherence was decreased by 7 ± 2% and 7 ± 4% for easy and difficult cues, respectively (mean ± SD across all subjects).

In both experiments, there were significant differences in response accuracy (experiment 1: *P* < 0.01; experiment 2: *P* < 0.01) and motion coherence (experiment 1: *P* < 0.01; experiment 2: *P* < 0.01) between easy and difficult cues (Fig. 6 *B* and *C*). The reaction time, measured from the fixation point offset to the start of the EMG activity in FCR for flexion movements, was significantly shorter for trials with easy cues (experiment 1: 173 ± 11 ms; experiment 2: 218 ± 20 ms) compared to trials with difficult cues (experiment 1: 226 ± 13 ms, *P* < 0.01; experiment 2: 247 ± 17 ms, *P* < 0.01; Fig. 6 *D* and *E*). Comparing behavioural results between experiment 1 and experiment 2 yielded no significant difference in response accuracy (easy cue: *P* = 0.2; difficult cue: *P* = 0.32), motion coherence (easy cue: *P* = 0.21; difficult cue: *P* = 0.37) or reaction time (easy cue: *P* = 0.08; difficult cue: *P* = 0.33).

Using single unit recordings in monkeys, Chandrasekaran *et al*. (2017) demonstrated that the firing rate of neurons in superficial layers during early discrimination of a visual cue was dependent on the stimulus difficulty, with lower difficulty stimuli associated with higher neuronal activity. According to these results in monkeys, we expected to find a similar modulation during early discrimination for the late part of the I1 volley (EFD +0.6 ms) in humans, i.e. higher conditioned H‐reflexes with low stimulus difficulty compared to those with high stimulus difficulty. No such changes were expected for the early part of the I1 volley (EFD 0 ms), which should depend on activity of deep layer neurons.

To test this hypothesis, we probed H‐reflex facilitation at the onset of the visual cue (tested in experiment 1), at 400 ms after cue onset (corresponding to the early discrimination phase according to Chandrasekaran *et al*. (2017), tested in experiment 1 and experiment 2), and at 300 ms after delay onset (tested in experiment 2).

**Figure 7:**
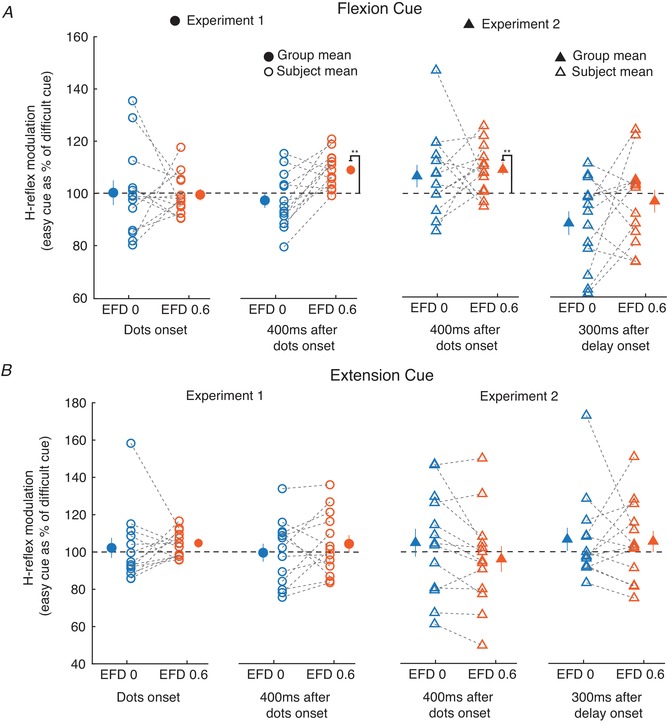
Conditioned H‐reflexes recorded during task performance *A* and *B*, changes in the facilitated FCR H‐reflex between easy compared to difficult cues for different stimulus timings relative to the task, and for EFD 0 and 0.6 ms. Results are shown separately for when the instructed direction was a flexion (*A*) or an extension (*B*). Filled circles and triangles represent grand mean ± SEM (^**^ *P* < 0.01). Open circles and triangles represent single subject mean. [Color figure can be viewed at wileyonlinelibrary.com]

Figure 7 shows the results. From all recorded trials, only trials were analysed where the cues instructed a flexion movement, because H‐reflexes in the flexor muscle FCR were measured (Fig. 7 *A*). Probing at visual cue onset yielded no differences in H‐reflex facilitation between easy and difficult cues, for both EFD 0 ms and EFD +0.6 ms (EFD 0 ms: *P* = 0.68; EFD +0.6 ms: *P* = 0.61; left plot of Fig. 7 *A*). At 400 ms after cue onset (middle two plots of Fig. 7 *A*), for EFD 0 ms there was again no difference in H‐reflex facilitation between easy and difficult cues (experiment 1: *P* = 0.56; experiment 2: *P* = 0.15). However, at EFD +0.6 ms, there was a robust difference, with greater H‐reflex facilitation with easy compared to difficult cues (*P* < 0.01 in both experiments 1 and 2). At 300 ms after delay onset (right plot in Fig. 7 *A*), there were no cue‐related differences for EFD +0.6 ms (*P* = 0.25). Differences for EFD 0 ms (*P* = 0.026) were not significant after *post hoc* (Bonferroni) correction.

In both experiment 1 and experiment 2, cue difficulty significantly influenced conditioned H‐reflexes at EFD +0.6 ms when probing took place 400 ms after visual cue onset; the conditioned H‐reflexes were larger for easy than for difficult cues. We wondered whether the conditioned H‐reflex was also influenced by the movement outcome (correctness of the choice). Therefore, all trials with cues instructing a flexion movement were selected. Correct (flexion) responses were compared with incorrect (extension) responses. This analysis yielded no effect of movement outcome (experiment 1: *P* = 0.24; experiment 2: *P* = 0.13).

Finally, we analysed trials where the visual cues instructed an extension movement (Fig. 7 *B*). This was of interest, because H‐reflex recordings were made from the FCR muscle. Analysing responses for instructed extension movements therefore allowed us to assess whether differences were widespread, or only seen if the recorded muscle was cued to move. In fact, for cued extension movements there were no differences in H‐reflex facilitation between easy and difficult cues for both 0 ms and +0.6 ms EFD, at each of the tested task epochs (experiment 1 cue onset: EFD 0 ms, *P* = 0.69; EFD +0.6 ms, *P* = 0.04; experiment 1 400 ms after cue onset: EFD 0 ms, *P* = 0.61; EFD +0.6 ms, *P* = 0.72; experiment 2 400 ms after the cue onset: EFD 0 ms, *P* = 0.93; EFD +0.6 ms, *P* = 0.26; experiment 2 300 ms after the delay onset: EFD 0 ms, *P* = 0.41; EFD +0.6 ms, *P* = 0.61). Thus, the reflex modulations with cue difficulty appear to be effector specific.

## Discussion

In this study, we used direct recordings in monkey to propose that non‐selective stimulation of the cortex generates a first indirect (I1) descending corticospinal volley composed of two distinct parts. The earliest part of the I1 volley appears to originate from stimulation of layer 5 neurons, which transynaptically activate adjacent corticospinal neurons via their basal proximal dendrites that reside in layer 5. Around 0.6 ms later, further corticospinal neurons can be recruited into the I1 volley by inputs from superficial dendrites in layers 2/3; the additional delay reflecting the slower EPSP rise time probably reflects the more distal location of these synaptic inputs on the corticospinal cell dendritic tree. However previous TMS and TES studies have not shown a bifid I1, which implies that the latencies of action potentials in the I1 spinal volley do not have a bimodal distribution. This is supported by the fact that in our monkey *in vivo* study in Fig. 5 *C*, the I1 latency is not bimodal but has a gradual transition from long to short as stimulus depth increases. TMS is likely to activate all layers simultaneously and will therefore elicit action potentials at all of the latencies show in Fig. 5 *C*. This could be due to the synapse‐to‐soma distances overlapping in the apical and basal dendritic trees.

Taken alone, this observation is of purely academic interest. Such small differences in the latency of activation after a highly artificial stimulus likely have no relevance for motor performance. However, when joined with a non‐invasive approach in humans, these observations open the possibility of measuring excitability changes of different cortical circuits and potentially dissociating layers in a straightforward way.

TMS in humans is likely to activate all layers in the motor cortex simultaneously (Di Lazzaro *et al*. 2008, 2012; Di Lazzaro & Ziemann, 2013). According to our *in vitro* findings, it might be expected that the I1 volley from TMS should display a double‐peaked appearance, because stimulation at different layers excites corticospinal neurons with different delays. However, previous TMS and TES studies do not show bifid I1 waveforms (Edgley *et al*. 1990; Di Lazzaro *et al*. 2008, 2018). In fact, our *in vivo* monkey results revealed a gradual, not step, transition from long to short I1 latency as the stimulus depth increased (Fig. 5 *C*). As TMS excites a wide area of cortex, the precise conduction times to the spinal cord will differ depending on the exact location of the cortical site activated; this dispersion will also act to smooth out any notch in the I1 waveform. It is thus perhaps unsurprising that reported I1 volleys following non‐invasive stimuli show a single peak.

In the human experiments H‐reflexes were used to dissect the early and late parts of the I1 (EFD 0 ms and EFD +0.6 ms). In the early phase of visual discrimination, we found a selective and cue‐related modulation of the late part. This is in line with previous monkey experiments and supports the idea that excitability changes of early and late parts of I1 likely reflect different cortical circuits.

It is important to point out our modifications to the method of PNS conditioning with TMS (Nielsen *et al*. 1993; Niemann *et al*. 2018) which allowed us to confine our conclusions to cortical circuits and exclude any spinal interference with high certainty. Firstly, we calculated RMS values of the first 0.5 ms of the EMG response from H‐reflex onset. This ensured that our measurements included only the earliest, monosynaptic component of the reflex (Burke, 2016). Secondly, we used small time intervals between TMS and median nerve stimuli (0.1 ms steps) in order to determine the earliest facilitation of the H‐reflex (EFD 0 ms) with high temporal precision. Thirdly, the ‘slowest’ conducting corticospinal volley we tested was only 0.6 ms delayed with respect to the fastest conducting volley. Taking all three modifications together, it is therefore highly unlikely that the excitability changes are mediated by spinal rather than cortical effects. Spinal circuits which could be affected by the earliest arriving corticospinal volley and influence spinal motoneuron responses at later corticospinal inputs would need more time (at least 1 ms) (Pierrot‐Deseilligny & Burke, 2005).

It is well known that the level of excitability of the motor cortex can influence the size of the descending volley elicited by TMS (Hess *et al*. 1986; Baker *et al*. 1995; Di Lazzaro *et al*. 1998, 2003). For indirect volleys, theoretically this could occur at two locations. Firstly, the TMS must excite an interposed interneuron directly. Assuming the stimulus activates this cell close to the cell body, the probability that an action potential is elicited within it will depend on the level of membrane depolarization, which will reflect the current balance of excitatory and inhibitory synaptic drive. Secondly, the interneuron must excite a corticospinal cell indirectly (trans‐synaptically). The synaptic currents will sum at the initial segment; whether they are sufficient to trigger an action potential will depend on the level of excitability of the corticospinal cell. The corticospinal volley amplitude must therefore reflect the excitability of both the interneurons and corticospinal neurons involved in I volley generation. Importantly, both the early and late part of the I1 volley must pass through the corticospinal neuron; changes in corticospinal cell excitability would therefore be expected to modulate both parts equally. Faced with a differential modulation of the early and late parts of I1 (EFD 0 ms and EFD +0.6 ms) in the dot motion discrimination task in humans, we conclude that this reflects a different modulation of the interneurons mediating each part.

Functional imaging can deliver sufficient spatial resolution to resolve deep and superficial layers in human motor cortex (Huber *et al*. 2017). However, such high‐resolution imaging is expensive and technically challenging. It cannot be easily applied to a wide range of motor tasks, due to the requirements for imaging stability and non‐magnetic manipulanda. In addition, functional imaging relies on the relatively sluggish BOLD response, which severely curtails temporal resolution. Many motor processes occur on a time scale unobservable with such methods: one example is the complex computation carried out in just 400 ms after dot onset in our task. The electrophysiological method described here can deliver measurements with very high temporal specificity relative to task performance.

Although our results point to the possibility of segregating layers in human M1 with H‐reflex conditioning by TMS, this interpretation needs to be treated with caution. Only direct measures in humans could unambiguously demonstrate that this is really possible. The currents induced by suprathreshold TMS are assumed to activate fast conducting corticospinal neurons in layer 5b trans‐synaptically; the earliest facilitation (EFD 0 ms) likely reflects this activation (Rothwell, 1997; Di Lazzaro *et al*. 2018). Further, TMS at suprathreshold intensity is assumed to excite neurons at different cortical layers simultaneously (Di Lazzaro *et al*. 2008, 2012; Di Lazzaro & Ziemann, 2013). This means that human and monkey experiments should be similar with regards to the anatomical origin and the timings of effects from the stimulations. However, depending on the placement of the coil with respect to the scalp and the coil orientation, it may be that structures other than fast conducting corticospinal neurons in M1 could have been targeted, such as axonal connections from the premotor to the motor cortex (Hamada *et al*. 2014; Volz *et al*. 2015). A contribution from such connections cannot be excluded. However, the close agreement between the data in monkey indicating a 0.6 ms time shift and the significant differences during task performance found in humans when we modify stimulus timing by this small value strongly suggests that our non‐invasive method is capable of resolving layer‐specific differences in excitability.

A further limitation of this technique is that not all possible results will admit unambiguous interpretations. Our primate data suggest that the earliest part of I1 can only arise from stimulation of deep (layer 5) cells. By contrast, later parts of I1 could have a contribution from both deep *and* superficial layers. In circumstances where we see changes in the early part of I1, it will thus be hard to interpret changes in the late part reliably. This was seen in our results for stimuli given 300 ms after delay onset (Fig. 7 *A* right plot), when there was a difficulty‐related modulation in the early part of I1 (EFD 0 ms) and a non‐significant similar modulation in the late part of I1 (EFD 0.6 ms). Unlike EFD 0 ms, the mechanistic interpretation of EFD 0.6 would be difficult. However, when we see no consistent changes in the early part of I1 (EFD 0 ms) but changes in later parts (EFD +0.6 ms), we believe that this provides good evidence that distinct cortical circuits have modulated their excitability. In support of this conclusion, our results were as expected from direct recordings of neurons in monkeys performing a discrimination task (Chandrasekaran *et al*. 2017; Peixoto *et al*. 2018).

One disadvantage of H‐reflex conditioning with TMS is that measurements can be very time‐consuming. It is necessary to find and maintain an H‐reflex in the FCR, locate the TMS hot spot, determine the threshold, and scan multiple ranges of conditioning intervals to locate EFD 0 ms prior to gathering the recordings of primary interest. This ‘preparation’ can easily take 2 h. It might be thought feasible to make the initial set‐up measurements on one day, and then perform the main experiment after a break; this could extend the approach to a wider range of subjects, e.g. to patients with movement or cognitive disorders. However, a problem that prevents such separate measurements is that any change in how the peripheral nerve is stimulated can affect the H‐reflex latency and thus the delay between TMS and PNS defining EFD 0 ms. We experienced this when subjects changed their arm position in the middle of the experiment. The slight modification of arm placement after a break could sometimes change the H‐reflex latency by 0.1–0.2 ms, necessitating re‐measurement of EFD 0 ms.

Although we have used this approach to study the upper limb, there is no reason why it could not also be deployed to investigate layer‐specific contributions to the control of the leg; indeed, this would be practically easier, as eliciting H‐reflexes in lower limb muscles such as soleus is generally straightforward. However, future investigations testing lower limb muscles with this method need to consider possible pitfalls concerning the possibility of evoking D volleys and not only I volleys around RMT (Houlden *et al*. 1999), and the reduced number of fast conducting monosynaptic projections in the lower leg compared to arm and hand muscles (Lemon, 2008).

In conclusion, using data from both monkey and human experiments we show that it is possible to measure excitability changes of different cortical circuits which likely reflect different cortical layers during a complex cognitive motor task, with high temporal resolution. We expect that this will open up new avenues for research into cognitive, motor and sensory processing in humans, both in health and in pathological states where changes in layer‐specific cortical circuits have been implicated, such as autism (Fang *et al*. 2014) and cortical dysplasia (Thom *et al*. 2005).

## Additional information

### Competing interests

Authors have no conflict of interest.

### Author contributions

A.K., W.X., P.W., C.L. and S.N.B. conceived and designed the research; A.K., W.X., P.W. and S.N.B. performed the experiments; A.K., W.X. and P.W. analysed the data; A.K., W.X., P.W., C.L. and S.N.B. interpreted the results of the experiments; A.K. and W.X. prepared the figures; C.L. and S.N.B. drafted the manuscript; A.K., W.X., P.W., C.L. and S.N.B. edited and revised the manuscript. All authors have read and approved the final version of this manuscript and agree to be accountable for all aspects of the work in ensuring that questions related to the accuracy or integrity of any part of the work are appropriately investigated and resolved. All persons designated as authors qualify for authorship, and all those who qualify for authorship are listed.

### Funding

This work was supported by the Deutsche Forschungsgemeinschaft (grant LE2744_10‐1 to C.L.) and by the Wellcome Trust (grant WT101002MA to S.N.B.).
